# The Timing of Drug Funding Announcements Relative to Elections: A Case Study Involving Dementia Medications

**DOI:** 10.1371/journal.pone.0056921

**Published:** 2013-02-27

**Authors:** Sudeep S. Gill, Neeraj Gupta, Chaim M. Bell, Paula A. Rochon, Peter C. Austin, Andreas Laupacis

**Affiliations:** 1 Department of Medicine, Queen's University, Kingston, Ontario, Canada; 2 Institute for Clinical Evaluative Sciences, Toronto, Ontario, Canada; 3 Department of Medicine and Institute of Health Policy, Management, and Evaluation, University of Toronto, Toronto, Ontario, Canada; 4 Department of Medicine, Mount Sinai Hospital, Toronto, Ontario, Canada; 5 Women's College Research Institute at Women's College Hospital, Toronto, Ontario, Canada; 6 Keenan Research Centre, Li Ka Shing Knowledge Institute of St. Michael's Hospital, Toronto, Ontario, Canada; “Mario Negri” Institute for Pharmacological Research, Italy

## Abstract

**Background:**

Following initial regulatory approval of prescription drugs, many factors may influence insurers and health systems when they decide whether to add these drugs to their formularies. The role of political pressures on drug funding announcements has received relatively little attention, and elections represent an especially powerful form of political pressure. We examined the temporal relationship between decisions to add one class of drugs to publicly funded formularies in Canada's ten provinces and elections in these jurisdictions.

**Methods:**

Dates of provincial formulary listings for cholinesterase inhibitors, which are drugs used to treat Alzheimer's disease and related dementias, were compared to the dates of provincial elections. Medical journal articles, media reports, and proceedings from provincial legislatures were reviewed to assemble information on the chronology of events. We tested whether there was a statistically significant increase in the probability of drug funding announcements within the 60-day intervals preceding provincial elections.

**Results:**

Decisions to fund the cholinesterase inhibitors were made over a nine-year span from 1999 to 2007 in the ten provinces. In four of ten provinces, the drugs were added to formularies in a time period closely preceding a provincial election (*P* = 0.032); funding announcements in these provinces were made between 2 and 47 days prior to elections. Statements made in provincial legislatures highlight the key role of political pressures in these funding announcements.

**Conclusions:**

Impending elections appeared to affect the timing of drug funding announcements in this case study. Despite an established structure for evidence-based decision-making, drug funding remains a complex process open to influence from many sources. Awareness of such influences is critical to maintain effective drug policy and public health decision-making.

## Introduction

More than two years after the historic passage of the *Patient Protection and Affordable Care Act* (ACA), ongoing debate over US health care reform has reinforced how profoundly politics can shape health policy. The results of the 2012 Presidential election will have a profound impact on the delivery of health care in the US for years to come [1].

Specific elements of health policy, however, are traditionally viewed as being relatively insulated from political influences. For example, insurers and health care systems in many countries provide formulary coverage for prescription medications and must balance rising costs against appropriate access to new treatments [2]–[4]. A number of agencies help to determine whether new drugs should be listed in drug formularies, and the principles that drive their decisions (such as evidence of effectiveness and safety, evidence of need, and cost implications) have been reviewed [2], [3], [5]. Although cost-effectiveness criteria are used to guide policy related to drug coverage in many countries, drug reimbursement decisions within publicly funded health care settings in the US largely exclude considerations of cost [4]. For example, the ACA specifically prohibits use of cost effectiveness thresholds to guide coverage decisions [6]. The emergence of very expensive treatments, such as biological treatments for cancer, has highlighted the issue of cost in making coverage decisions [7], [8].

The Canada Health Act supports a near-universal system of health care across the country's ten provinces, although each province makes its own decisions about which prescription drugs it will cover (**Appendix S1**). Despite well-established principles to guide decisions, significant differences have been observed in formulary coverage of drugs across Canada, with the timing of drug additions onto different provincial formularies varying in some cases by years [9], [10].

External forces may help to explain these variations. For example, some have speculated that undue political pressures may have influenced certain drug formulary decisions [11], [12]; however, this theory has not been formally tested. Capturing the many nuances of political influence can be challenging, but elections are easily quantified and represent a particularly acute form of political pressure. To assess the relationship between funding decisions and this form of political pressure, we examined the association between the timing of provincial funding announcements for a class of drugs known as the cholinesterase inhibitors and the timing of elections in these provinces.

Alzheimer's disease is the sixth leading cause of death in the US, and there are still no effective treatments to prevent, halt or reverse this condition [13], [14]. Cholinesterase inhibitors were the first drug treatments approved for Alzheimer's disease and related dementias. Health Canada approved donepezil (Aricept) in 1997, rivastigmine (Exelon) in 2000, and galantamine (Razadyne, Reminyl) in 2001. Cholinesterase inhibitor use is widespread, with global sales of donepezil reaching $4.4 billion dollars in 2010 [15]–[18]. In Ontario, cholinesterase inhibitor prescriptions grew dramatically between 2000 and 2011 (**Figure S1**).

We chose to focus on cholinesterase inhibitors for this case study because they represented the first major therapeutic advance for dementia. Several debates about their clinical and cost effectiveness arose between the premarketing stage and the recent arrival of generic formulations signaling the final stage of their product life cycle [17]–[19]. A recent trial confirms the clinically marginal benefits of continued cholinesterase inhibitor treatment in patients with more advanced dementia [20], and there is now general consensus that these drugs possess modest efficacy [17]–[19].

## Methods

We reviewed reports that covered the regulatory approval of cholinesterase inhibitors by Health Canada, the efforts of pharmaceutical manufacturers and patient advocacy groups to have these drugs reimbursed by provincial drug formularies, and announcements from provincial governments about formulary coverage for these medications. Canada's approach to drug approval and reimbursement is described in **Appendix S1**. We did not examine drug formulary decision-making or elections in Canada's three territories (Nunavut, the Northwest Territories, and Yukon) because of their relatively small population and their unique demographic profile. Much of the population of these territories is First Nations and they receive prescription medications from special federal and territorial programs. We performed a comprehensive search of public agencies, media outlets, and Google News, to identify news media reports with the dates of important funding announcements, and the PubMed database to identify relevant medical journal articles. We then reviewed proceedings from provincial legislatures that included transcripts of discussions about public reimbursement for the cholinesterase inhibitors. Whenever possible, we determined the earliest date that funding decisions were announced. We identified dates of provincial elections from official elections websites for each province (e.g., http://www.electionsmanitoba.ca/).

### Data analysis

We identified drug funding announcements for cholinesterase inhibitors made in the 60-day period preceding a provincial election. A 60-day period was chosen as this is the usual time interval between the announcement of a provincial election and voting, and represents the time when political parties most actively campaign to gain voter support. To determine whether there was a statistically significant change in the probability of drug funding announcements released within the 60-day interval prior to a provincial election, we conducted a one sample test of a binomial probability. The null hypothesis was that the probability of drug funding announcements occurring within 60 days prior to provincial elections was 0.0493; that is, 4.93% of funding announcements (roughly one in 20) would have been expected by chance alone in the 60-day intervals preceding elections. This expected probability was calculated by determining the proportion of time that provinces spent in 60-day pre-election periods between January 1, 1999 and December 31, 2007. These dates were chosen because cholinesterase inhibitors were approved between June 1999 and October 2007 in the ten provinces. A one sample test of a binomial proportion was used to compare the observed proportion of funding announcements in the 60-day intervals preceding elections to the null hypothesis value of 0.0493. The threshold for statistical significance was *P*<0.05. Further details are provided with **Appendix S2**.

## Results

Cholinesterase inhibitors were added to formularies in all ten Canadian provinces between June 1999 and October 2007. None of these decisions were modified or reversed when followed up to September 2012. Four of the ten drug funding announcements occurred within 60-day intervals preceding provincial elections (**Table 1**). This observed proportion was significantly greater than that expected by chance (*P* = 0.032).

**Table 1:** Timeline detailing announcements of cholinesterase inhibitor inclusion onto provincial formularies and dates of provincial elections^a^.

Province	Cholinesterase Inhibitors Added to Provincial Formulary	Date of Closest Provincial Election	Incumbent Party	Winner of Election	Time Between Announcement and Closest Election (days^b^)
Ontario	1 June 1999	3 June 1999	PC	PC	−2
Manitoba	11 August 1999	21 September 1999	PC	NDP	−41
Alberta	1 December 1999	12 March 2001	PC	PC	−467
Quebec	16 May 2000	20 November 1998	PQ	PQ	+543
Saskatchewan	24 October 2000	16 September 1999	NDP	NDP^c^	+404
Nova Scotia	19 June 2003	5 August 2003	PC	PC^c^	−47
New Brunswick	21 August 2003 (announcement of funding to begin on 1 September 2003)	9 June 2003	PC	PC	+73
Newfoundland and Labrador	18 September 2003 (initial plan to fund drugs announced^d^)	21 October 2003	L	PC	−33
	30 March 2006 (announcement of funding to begin on 1 September 2006)	9 October 2007	PC	PC	−528
Prince Edward Island	22 July 2005	29 September 2003	PC	PC	+662
British Columbia	4 October 2007 (announcement of funding to begin on 22 October 2007)	12 May 2009	L	L	−586

Abbreviations: L, Liberal; NDP, New Democratic Party; PC, Progressive Conservative; PQ, Parti Quebecois.

a Provinces are listed in order of when cholinesterase inhibitors were added to provincial drug formulary. In four provinces (Ontario, Manitoba, Nova Scotia, and Newfoundland and Labrador), announcements of drug formulary coverage were made within the 60-day period preceding a provincial election.

b For the time between announcement and closest election, the number of days is given as either the numbers of days preceding an election (a negative number, e.g. −2 days) or the number of days following an election (a positive number, e.g. +73 days).

c Minority government.

d Decision to add cholinesterase inhibitors to Newfoundland and Labrador drug formulary announced by Liberal government on 18 September 2003 was delayed, after Liberal government defeated by Progressive Conservatives in October 2003 election.

The four affected provinces were Ontario, Manitoba, Nova Scotia, and Newfoundland and Labrador. Detail regarding the chronology and context of events for each of these provinces is provided below and in **Figures S2, S3, and S4**.

### Ontario

Health Canada approved donepezil in August 1997. Despite enthusiasm about the availability of a dementia treatment, some clinicians initially voiced concerns about offering it to their patients because only one randomized controlled trial (RCT) evaluating this drug had been published by that time [21], [22]. Two further RCTs were published in January and May 1998 [23], [24], and many more RCTs and reviews evaluating the cholinesterase inhibitors have been published since then [17], [20]. A newspaper report on 18 July 1998 highlighted requests from a dementia specialist and the Alzheimer Society to have donepezil added to Ontario's drug formulary [25]. This report quoted a spokesperson for Ontario's health ministry, who stated that the drug was still under review. Another newspaper article on 13 August 1998 cited complaints from the Pharmaceutical Manufacturers Association of Canada about the slow uptake of new drugs such as donepezil onto Ontario's formulary [26]. The article quoted a response from Ontario's then-premier: “There are drug companies who would like to see their products on the market earlier. And of course they apply that pressure.” The premier also stated, “…[the government has] an obligation to ensure the effectiveness of those drugs, [part of which includes a cost-benefit analysis].”

On 1 June 1999, donepezil was added to Ontario's drug benefit program, making Ontario the first province in Canada to cover a cholinesterase inhibitor through a publicly funded drug plan [27]. The provincial election took place 2 days later, on 3 June 1999. The incumbent party won this election (**Table 1**).

### Manitoba

The Manitoba government's decision to place donepezil on the provincial formulary followed soon after the decision in Ontario, making them the second province to cover this drug. Initially, there appeared to be reluctance to fund donepezil; a news report from 28 July 1999 quoted a spokesperson for the health ministry stating that, “there is not the type of evidence to provide full coverage for the drug,” despite the decision in June to fund donepezil in neighboring Ontario [28]. Less than a month later, on 11 August 1999, the Manitoba government announced the addition of donepezil to their provincial drug formulary [29]. The provincial election followed on 21 September 1999. The incumbent party lost this election but the drug funding decision was not altered.

### Nova Scotia

Several discussions related to coverage of the cholinesterase inhibitors took place in the Nova Scotia legislature during 2002. Themes emerging from these discussions included calls from opposition party members to add the drugs to the formulary as had been done in five other provinces by that time (**Table 1**), and government members responding that regular review by the province's formulary review committee did not find sufficient evidence to support coverage of what was viewed as an expensive class of drugs. For example, on 23 May 2002, an opposition member asked why the government was “dragging its heels” in making a funding decision. The health minister responded by saying the ministry's advisory committee, which considers “…among other things, cost-effectiveness”, did not think the evidence available at that point supported the addition of the cholinesterase inhibitors to the provincial formulary [30]. On 6 November 2002 during a session of the Public Accounts Committee devoted to pharmaceutical coverage, the deputy minister of health was asked why the cholinesterase inhibitors had not yet been added to the province's formulary [31]. The deputy minister responded that these drugs were under review, but concerns lingered regarding their expense and the fact that they appeared to be effective in only 25–40% of patients who received them. When challenged with the fact that five other provinces had already added these drugs to their formularies (“What particular evidence…are you looking for that the other provinces have accepted that you don't accept?”), the deputy minister responded, “…in the bulk of those jurisdictions they were listed in the months preceding provincial elections…The general sense is that there was some potential political expediency with those decisions” [31]. Later that day, another discussion about coverage of the cholinesterase inhibitors occurred during the general assembly and the health minister stated: “Mr. Speaker, the Formulary Review Committee in Nova Scotia is an arm's-length body, and I can tell the House that they make the decisions on evidence and as Minister of Health I have not interfered with one of their recommendations in the time that I've been in office” [32].

On 19 June 2003, the government announced that it would cover the cholinesterase inhibitors on the provincial formulary, making Nova Scotia the sixth province to list these drugs [33]. The provincial election took place on 5 August 2003. The incumbent party was re-elected, albeit as a minority government.

### Newfoundland and Labrador

Proceedings from the provincial legislature on 1 December 1999 show some of the first government debate about coverage of cholinesterase inhibitors in Newfoundland and Labrador [34]. In response to questions from the opposition party, the minister of health and community services stated that coverage for donepezil was under review and challenged the opposition member's assertion that availability of donepezil might lead to reduced costs by delaying nursing home placement.

On 4 April 2000, the legislative proceedings again document a discussion about funding for donepezil [35]. An opposition member called on the health minister to “…do what they are doing in Ontario and Manitoba and approve a twelve week prescription trial for this drug.” In response, the health minister stated:

I just happened to come from a meeting of Health Ministers… It is interesting that he mentioned Ontario and Manitoba, because I happened to ask both those ministers if they had actually approved Aricept on the basis of clinical evidence. Their answer was, no.

The answers given were these: they suggested that they would have been better advised to wait for clinical evidence from those other than the company trying to sell the drug, which is what we are waiting for; and that in Ontario they said they did it because there was public pressure and they had the money, which is not our case; and in Manitoba they did it because it was announced during an election.

They admitted that in both cases neither of them did it on the basis of evidence…[35]

On 18 September 2003, the government of Newfoundland and Labrador announced that the cholinesterase inhibitors would be added to the provincial formulary as part of a new seniors' drug program [36]. However, when the provincial election took place a month later on 21 October 2003, a new party was elected and coverage for the cholinesterase inhibitors was deferred.

On 21 April 2005, the new health minister defended this delay by citing the AD2000 study, a British RCT published in June 2004 that suggested treatment with donepezil was associated with no significant improvements in rates of disability or institutionalization, caregiver burden, or overall costs [37], [38]. (Although interpretation of the AD2000 trial generated considerable debate when it was published, there is now consensus that the benefits of treatment with cholinesterase inhibitors are generally modest [17], [20].) During the announcement of the provincial budget in legislature on 30 March 2006, the health minister revealed that the cholinesterase inhibitors would be added to the formulary beginning in September 2006 [39]. This was announced as part of a broad package of new funding in health care that was attributed to economic growth in the province.

## Discussion

Canada's ten provinces announced coverage for cholinesterase inhibitors over a nine-year period stretching from 1999 to 2007. This marked variation likely reflects the influence of various factors including uncertainty about the evidence base supporting the use of these drugs, media reporting, lobbying efforts by pharmaceutical manufacturers and patient advocacy groups, and competing priorities that were unique to each province. The complex interplay of these multiple influences shifts continuously during the life cycle of any drug, from its initial introduction onto the market with limited information from RCTs, through growing clinical experience and postmarketing surveillance, until well after the availability of generic formulations of the product [40]. Beyond this dynamic interaction of multiple influences, we found evidence in this example of a relationship between drug funding announcements and political pressure in the form of impending elections. In our case study, four of ten provinces added cholinesterase inhibitors to their formularies in the 60-day period preceding an election, including the first two provinces to list these drugs. At least two politicians stated that the timing of announcements was influenced by political pressures [31], [35].

These findings provide an additional explanation for the variability in drug coverage observed in different jurisdictions [9], [10]. Qualities specific to individual provinces must also be considered: for example, the size of the population likely to require the drug, and the funds available for health care in a particular budget cycle [5], [41], [42].

The case of the cholinesterase inhibitors also exemplifies the challenges involved in deciding when sufficient evidence has accumulated to warrant formulary coverage for expensive new drug treatments [7], [8], [43]. Two provinces initially cited the disappointing results of the AD2000 trial as a reason for rejecting formulary coverage for these drugs [37], [44]; subsequent decisions in both provinces to fund the drugs supports the notion that influences other than scientific evidence may trigger drug policy decisions.

Our focus on cholinesterase inhibitors strengthened this study for several reasons. First, this drug class included the first approved drug treatments for Alzheimer's disease, a common and devastating illness. There would be less interest and political gain in the approval of a drug that was simply an addition to an existing and previously populated drug class (i.e., a “me too” drug) or one that targeted a disease with a less fearsome public profile. Second, the cholinesterase inhibitors have nearly completed the drug life cycle from pre-approval to the recent arrival of generic formulations. This allowed us to take a long-term view and thoroughly analyze the funding decisions that were made along the way. By comparison, examination of newly approved drugs is often difficult given uncertainty about their long-term effectiveness and safety. Drug formulary decisions are usually deferred until such evidence becomes available. Finally, the cholinesterase inhibitors are still relevant, as they remain the most commonly prescribed medications for Alzheimer's disease and related dementias. Recent publications about these drugs include a commentary discussing the strategies undertaken to extend the patent life of donepezil [45], and a RCT called DOMINO that demonstrated marginal benefits associated with continuing donepezil in people with moderate to severe dementia [20].

Other literature about the relationship between political forces and health policy decisions merits consideration. Some reports have speculated about the link between elections and the timing of drug funding announcements, but have not attempted to quantify this association [46]. Examples involving the human papillomavirus (HPV) vaccine Gardasil [11], [47] and the adjuvant breast cancer treatment trastuzumab (Herceptin) [12] demonstrate that drug policy decision-making requires balancing evidence of clinical effectiveness, safety, and cost-effectiveness on the one hand, and external factors – such as media reports, lobbying by various parties, and political interests – on the other. Additional factors such as the need for legislators to make decisions quickly despite limited expertise may also affect the timing of decisions [48].

The link we show here between upcoming elections and drug funding announcements raises concern because the external pressures and stringent deadlines involved in elections may adversely affect health policy decisions. To illustrate this principle, consider the drug review deadlines established at the Food and Drug Administration (FDA) after the Prescription Drug User Fee Act (PDUFA) was enacted. As compared with drugs approved at other times, drugs approved in the two months before their PDUFA deadlines were more likely to be withdrawn for safety reasons, more likely to carry a subsequent black-box warning, and more likely to have one or more dosage forms voluntarily discontinued by the manufacturer [49].

Politics has a necessary and fundamental role in shaping health policy decisions [50], but safeguards are needed to prevent undue political maneuvering by self-interested stakeholders. An example of the potential consequences of political interference on healthcare legislation involves the introduction of Medicare Part D, the US program that subsidizes the costs of prescription drugs for seniors and the disabled [51]. Part D was enacted as a component of the Medicare Modernization Act of 2003. Some members of Congress initially proposed authorizing the federal government to negotiate for lower drug prices. However, Congressional leaders (some of whom later left their government jobs to become lobbyists employed by the pharmaceutical industry [52]) included a clause in the final bill that prohibited the government from negotiating with pharmaceutical companies. In contrast, government is allowed to negotiate drug prices for Medicaid and programs sponsored by the Department of Veterans Affairs, allowing these programs to pay less for drugs than Medicare. Estimates suggest that extension of existing price setting mechanisms to Medicare Part D could result in annual savings of over $20 billion [53].

Our study has important limitations. First, it is based on evaluation of a single drug class, and it is certainly not generalizable to all medications. However, we have argued above for the value of a case study approach and for our focus on the cholinesterase inhibitors. Second, we had limited access to information on the impact of lobbying efforts by manufacturers and patient advocacy groups on governments as they made funding decisions. Negotiations between manufacturers and government can sometimes lead to incentives (e.g., cost sharing) that help to shape the final funding decision. Third, it is important to distinguish the timing of a public announcement of drug funding from the earlier time that the funding decision was reached. Government officials in Canada typically make such decisions after considering reimbursement recommendations from a formulary review committee, but information about the timing and content of recommendations made by formulary committees and the subsequent decisions reached by government are often not made public. We only had information on the timing of public announcements. In this context, a strong case can be made for increased transparency at all steps of the drug approval and reimbursement process [54].

### Conclusions

Although undue intrusion from political factors can distort sound health care policy, it is unrealistic to assume scientific evidence will be used as the sole criterion to guide policy decisions. These decisions also have to consider the interests and values of the public who elect government officials [55]. Several strategies have been put forward to ensure scientific evidence is effective in shaping health policy. Concise and relevant summaries of scientific research, transparency of policy decision-making at all stages, and close contact between scientists and policymakers can promote informed health policy decisions. Specialized knowledge brokers and translational scientists can act as bridges between scientists and policymakers to increase the likelihood that relevant scientific data is incorporated into health policy [56]. Training policy makers to take an evidence-based approach can also facilitate the effective use of the best evidence in healthcare [48].

Such approaches may help to shape effective health policy, and many jurisdictions have established processes to encourage an evidence-based approach to drug formulary decision-making. Nonetheless, drug funding decisions remain open to influence from many sources. The results of our case study warrant concern because politically expedient decisions may have been made before some impending elections. Awareness of such influences is critical to maintain effective drug policy and public health decision-making in the future.

## Supporting Information

Appendix S1 **Canada's drug approval process.**
(DOC)

Appendix S2 **Calculation of expected probability for null hypothesis.**
(DOC)

Figure S1 **Total cost and number of drug claims for cholinesterase inhibitors between January 2000 and December 2011 in Ontario, Canada.**
(DOC)

Figure S2 **Timeline detailing elections and approvals of cholinesterase inhibitors in four provinces.**
(DOC)

Figure S3 **Map of Canada detailing where and when cholinesterase inhibitors were first announced for inclusion on provincial drug formularies.**
(DOC)

Figure S4 **The time to drug funding announcements for cholinesterase inhibitors, defined as the number of days which elapsed following Ontario's drug funding announcement in June 1999.**
(DOC)
