# Supplementary material for: The Timing of Drug Funding Announcements Relative to Elections: A Case Study Involving Dementia Medications
Source: PLoS One. 2013 Feb 27;8(2):e56921. doi: 10.1371/journal.pone.0056921 (PMC3584056; doi:10.1371/journal.pone.0056921)
Supplement: Appendix S1 — Canada's drug approval process. (DOC) [file pone.0056921.s001.doc]

**Appendix S1. Canada’s Drug Approval Process**

Background may be useful for readers unfamiliar with the Canadian approach to prescription drug approval and reimbursement. The Therapeutic Products Directorate of Health Canada is responsible for the approval of new drugs for sale in Canada, analogous to the role of the Center for Drug Evaluation and Research (CDER) division of the Food and Drug Administration (FDA) in the US. Access to drug treatments in Canada is facilitated for some patient groups (e.g., those over age 65 years, those receiving social assistance) by coverage on publicly funded formularies. Each of Canada’s ten provinces maintains a separate drug formulary. The process of approving new drugs for inclusion on a provincial formulary involves a review of the drug’s clinical effectiveness and cost-effectiveness by a formulary review committee, and then approval of the committee’s reimbursement recommendation by an executive officer representing the provincial government. Several different formulary review committees exist, including those that are provincial (e.g., Ontario’s Committee to Evaluate Drugs), and those that are regional (e.g., the Atlantic Expert Advisory Committee). In 2003, the Common Drug Review was created to provide complementary cross-national reimbursement recommendations to participating federal, provincial, and territorial drug plans. Others have described the review process in greater detail.^1-6^

**References for Appendix S1**

1. Laupacis A. (2002) Inclusion of drugs in provincial drug benefit programs: who is making these decisions, and are they the right ones? CMAJ 166(1):44-47.
2. Laupacis A. (2006) On bias and transparency in the development of influential recommendations. CMAJ 174(3):335-336.
3. Canadian Agency for Drugs and Technologies in Health Common Drug Review (2012) About the Common Drug Review. Available: <http://www.cadth.ca/en/products/cdr/cdr-overview>. Accessed 25 September 2012.
4. McMahon M, Morgan S, Mitton C. (2006) The Common Drug Review: A NICE start for Canada? Health Policy 77(3):339-351.
5. Tierney M, Manns B, Members of the Canadian Expert Drug Advisory Committee. (2008) Optimizing the use of prescription drugs in Canada through the Common Drug Review. CMAJ 178(4):432-435.
6. Clement FM, Harris A, Li JJ, Yong K, Lee KM, et al. (2009) Using effectiveness and cost-effectiveness to make drug coverage decisions: a comparison of Britain, Australia, and Canada. JAMA 302(13):1437-1443.
