# Supplementary material for: The Timing of Drug Funding Announcements Relative to Elections: A Case Study Involving Dementia Medications
Source: PLoS One. 2013 Feb 27;8(2):e56921. doi: 10.1371/journal.pone.0056921 (PMC3584056; doi:10.1371/journal.pone.0056921)
Supplement: Appendix S2 — Calculation of expected probability for null hypothesis. (DOC) [file pone.0056921.s002.doc]

**Appendix S2.** Calculation of Expected Probability for Null Hypothesis.

| **Province** | **Number of elections during study period^a^** | **Number of days prior to election (60 days/election)** | **Proportion of time spent in 60-day intervals preceding elections during study period^a^** |
| --- | --- | --- | --- |
| Ontario | 3 | 180 | 0.0548 |
| Manitoba | 3 | 180 | 0.0548 |
| Alberta | 2 | 120 | 0.0365 |
| Quebec | 2 | 120 | 0.0365 |
| Saskatchewan | 3 | 180 | 0.0548 |
| Nova Scotia | 3 | 180 | 0.0548 |
| New Brunswick | 3 | 180 | 0.0548 |
| Newfoundland | 3 | 180 | 0.0548 |
| Prince Edward Island | 3 | 180 | 0.0548 |
| British Columbia | 2 | 120 | 0.0365 |
| Totals | 27 | 1620 | 0.4931 |
| **Expected probability for null hypothesis (mean proportion of time spent in 60-day intervals preceding elections divided by 10, i.e. total number of provinces)** | | | **0.0493** |

^a^ Study period was between January 1, 1999 and December 31, 2007 (i.e., 9 x 365 = 3285 days). These dates were chosen because cholinesterase inhibitors were approved between June 1999 and October 2007 in the ten provinces.

We used the following approach to determine the expected probability for the null hypothesis. The expected probability was the proportion of time, on average, that provinces spent in the 60-day intervals preceding elections between January 1, 1999 and December 31, 2007. For each province, we identified the number of provincial elections that took place during the nine-year study period (3285 days), and then determined the total number of days spent in 60-day intervals prior to elections in each province. The mean proportion of time spent in these 60-day intervals was then calculated. The expected probability was 0.0493; that is, 4.93% of funding announcements (roughly one in 20) would be expected in the 60-day intervals before elections if they were randomly scattered throughout the study time period.

This expected probability of 4.93% for the null hypothesis was then compared to the observed probability of funding announcements in the 60-day intervals preceding elections. *P* < 0.05 was considered the threshold for statistical significance.

For calculation of both expected and observed probabilities, we included data from all 27 elections that occurred in the 10 provinces during the nine-year study period. For both calculations, we did not exclude any elections that took place following funding announcements (i.e. no attempt was made to censor events or model them in a time-to-event fashion).
