# Supplementary material for: The Timing of Drug Funding Announcements Relative to Elections: A Case Study Involving Dementia Medications
Source: PLoS One. 2013 Feb 27;8(2):e56921. doi: 10.1371/journal.pone.0056921 (PMC3584056; doi:10.1371/journal.pone.0056921)
Supplement: Figure S1 — Total cost and number of drug claims for cholinesterase inhibitors between January 2000 and December 2011 in Ontario, Canada. (DOC) [file pone.0056921.s003.doc]

**Figure S1.** **Total cost and number of drug claims for cholinesterase inhibitors between January 2000 and December 2011 in Ontario, Canada.** Total cost (in millions, gray bars) and number of drug claims (in thousands, black line) for cholinesterase inhibitors (ChEIs) for each quarter (i.e., 3-month period) between January 1, 2000 and December 31, 2011 for the provincial drug benefit formulary in Ontario, Canada. Overall costs for ChEIs listed in the formulary have started to fall despite relatively stable numbers of claims because of the introduction of generic versions of rivastigmine (beginning in the fourth quarter of 2009) and galantamine (beginning in the first quarter of 2011). Data are from the Institute for Clinical Evaluative Sciences.
