# Supplementary material for: The Timing of Drug Funding Announcements Relative to Elections: A Case Study Involving Dementia Medications
Source: PLoS One. 2013 Feb 27;8(2):e56921. doi: 10.1371/journal.pone.0056921 (PMC3584056; doi:10.1371/journal.pone.0056921)
Supplement: Figure S2 — Timeline detailing elections and approvals of cholinesterase inhibitors in four provinces. (DOC) [file pone.0056921.s004.doc]

**Figure S2.** **Timeline detailing elections and approvals of cholinesterase inhibitors in four provinces.** Timeline detailing provincial elections and provincial approval of cholinesterase inhibitors (donepezil, rivastigmine and galantamine) in Ontario (red arrows), Manitoba (blue arrows), Newfoundland and Labrador (green arrows) and Nova Scotia (purple arrows). ChEI, cholinesterase inhibitor; RCT, randomized controlled trial.
