# Supplementary material for: The Timing of Drug Funding Announcements Relative to Elections: A Case Study Involving Dementia Medications
Source: PLoS One. 2013 Feb 27;8(2):e56921. doi: 10.1371/journal.pone.0056921 (PMC3584056; doi:10.1371/journal.pone.0056921)
Supplement: Figure S3 — Map of Canada detailing where and when cholinesterase inhibitors were first announced for inclusion on provincial drug formularies. (DOC) [file pone.0056921.s005.doc]

**Figure S3.** Map of Canada detailing where and when cholinesterase inhibitors were first announced for inclusion on provincial drug formularies.

The provinces are Alberta (AB), British Columbia (BC), Manitoba (MB), New Brunswick (NB), Newfoundland and Labrador (NL), Nova Scotia (NS), Ontario (ON), Prince Edward Island (PE), Quebec (QC), and Saskatchewan (SK). Canada’s three territories are Nunavut (NU), Northwest Territories (NT), and Yukon (YT). The timing of funding decisions has an apparent “ripple effect” with spread from central provinces like Ontario towards eastern and western provinces.
