# Supplementary material for: The Timing of Drug Funding Announcements Relative to Elections: A Case Study Involving Dementia Medications
Source: PLoS One. 2013 Feb 27;8(2):e56921. doi: 10.1371/journal.pone.0056921 (PMC3584056; doi:10.1371/journal.pone.0056921)
Supplement: Figure S4 — The time to drug funding announcements for cholinesterase inhibitors, defined as the number of days which elapsed following Ontario's drug funding announcement in June 1999. (DOC) [file pone.0056921.s006.doc]

**Figure S4.** The time to drug funding announcements for cholinesterase inhibitors, defined as the number of days which elapsed following Ontario’s drug funding announcement in June 1999.

Provinces are grouped by their distance from Ontario: 1) provinces adjacent to Ontario are Quebec (QC) and Manitoba (MB); 2) mid-distant provinces include Saskatchewan (SK) and Alberta (AB); and 3) distant provinces include British Columbia (BC) and the four maritime provinces of Newfoundland (NF), New Brunswick (NB), Nova Scotia (NS), and Prince Edward Island (PE). Data are plotted for each province along with mean (horizontal line) for each of the three groups of provinces.

An apparent “ripple effect” is observed, with provinces closest to Ontario taking less time to make their own drug funding announcements.
